# Molecular mechanisms of plant freezing tolerance: from cold signal perception to adaptive responses

**DOI:** 10.3389/fpls.2026.1850456

**Published:** 2026-05-28

**Authors:** Mingjie Wei, Xinyuan Liu, Yawen Wang, Guozhen Yan, Qian Li, Cheng Tang, Feng Shi

**Affiliations:** 1 College of Life Sciences, Shihezi University, Shihezi, China; 2 College of Urban and Environmental Sciences, Shihezi University, Shihezi, China; 3 College of Agriculture, Shihezi University, Shihezi, China

**Keywords:** Ca^2+^–ROS signaling, cold sensing, epigenetic regulation, freezing stress, *ICE–CBF–COR* axis, kinase cascade, membrane lipid remodeling, osmotic adjustment

## Abstract

Freezing stress poses a threat to plant survival and crop production; therefore, understanding the molecular basis of freezing tolerance and broader cold hardiness is essential for developing cold-hardy crop varieties. This review summarizes molecular responses to freezing, proposes a causal framework from perception to signaling, phenotypes, and applications, and compiles genetic evidence for early signals (Ca^2+^, ROS, kinases). We outline the *ICE–CBF–COR* transcriptional network and its multi-layered regulation, and integrate protective metabolic modules (osmotic adjustment, antioxidant defense, membrane lipid remodeling, protective proteins). Phytohormones and epigenetic and non-coding RNA regulation are also discussed, with an emphasis on cold-stress memory. Finally, we discuss strategies to improve freezing tolerance and cold hardiness through natural variation, genome editing, and cold-inducible expression systems, thereby informing molecular breeding and rigorous phenotyping.

## 1 Introduction

Low-temperature stress is divided into chilling (non-freezing low temperatures, typically approximately 0–15°C) and freezing (<0 °C) (Wu et al., 2022; Shomo et al., 2024). Chilling primarily depresses cellular metabolism and reduces membrane fluidity, thereby reversibly inhibiting growth (Kidokoro et al., 2022; Kim et al., 2024). In addition, freezing causes ice crystals to pierce cell membranes both inside and outside cells, resulting in cellular dehydration; if this happens directly, it can lead to death (Qiu et al., 2024; Charrier et al., 2026). In crop plants, cold stress can compromise several key developmental and physiological processes, including germination, seedling establishment, vegetative growth, photosynthetic performance, reproductive development, grain or fruit set, and ultimately yield stability (Burnett and Kromdijk, 2022; Dong et al., 2023; Zhou et al., 2023; Dong et al., 2025; Li et al., 2026; Yuan et al., 2026). At the agronomic scale, such responses are often reflected in delayed emergence, weaker canopy establishment, limited carbon assimilation, pollen or anther damage, reduced reproductive success, and less stable productivity during chilling or freezing events. The resulting injury is typically linked to membrane leakage, oxidative stress, disturbed water relations, and progressive cellular dehydration (Kidokoro et al., 2022; Delapierre et al., 2024; Kim et al., 2024). Several genes, loci, and regulatory modules have been implicated in crop cold tolerance, such as *COLD1*, *OsCRT3–OsCIPK7*, *CTB3*/*CTB5*, *STAYGREEN*, *ZmICE1*/*ZmHSF21*, *TaSAMT1*, as well as the broadly conserved *ICE1–CBF*/*DREB1–COR* regulatory module (Hwarari et al., 2022; Jiang et al., 2022; Dong et al., 2023; Guo et al., 2023; Zhou et al., 2023; Chu et al., 2024; Gao et al., 2024a; Guo et al., 2025; Li et al., 2025). Most temperate plants can develop freezing tolerance through cold acclimation; that is, exposure to non-freezing low temperatures before freezing episodes enhances their subsequent capacity to withstand freezing stress (Sugita et al., 2024; Takahashi et al., 2024). Acclimation describes inducible and often reversible physiological, biochemical, and molecular adjustments that develop within an individual plant after exposure to low, non-freezing temperatures. Adaptation, in contrast, refers to longer-term, heritable evolutionary changes that enhance performance in cold environments at the population or species level (Shikata et al., 2025; Wang et al., 2025). At the ecological or evolutionary level, plant adaptation to subzero environments generally involves two major strategies: freezing tolerance and freeze avoidance (Kane and McAdam, 2025). In this review, cold hardiness and cold/freezing resistance are used as broad umbrella terms to describe the overall capacity of plants to limit low-temperature injury at the whole-plant, organ, or cellular scale. By comparison, freezing tolerance refers more narrowly to the ability to survive extracellular or tissue ice formation through protective mechanisms induced during acclimation, whereas freeze avoidance describes strategies that delay or prevent internal ice formation, such as supercooling. Freezing-tolerant species, such as winter wheat and European aspen, can survive tissue freezing through dehydration-protection mechanisms and antifreeze proteins (Mustamin et al., 2023; Arroyo-Mosso et al., 2025; Chen et al., 2025; Johnson et al., 2025). Freeze-avoidant species, such as some evergreen woody plants, reduce internal ice formation by means of supercooling and other processes (Charra-Vaskou et al., 2023; Ingram et al., 2025). To improve agricultural cold hardiness, breeders can develop cold-hardy or freezing-tolerant crop varieties, while management practices that prolong the pre-winter acclimation period or apply insulating covers can reduce low-temperature injury (Boekee et al., 2023; Cano-Ramirez et al., 2023; Zhu et al., 2023; Dong et al., 2025).

**Figure 1:**
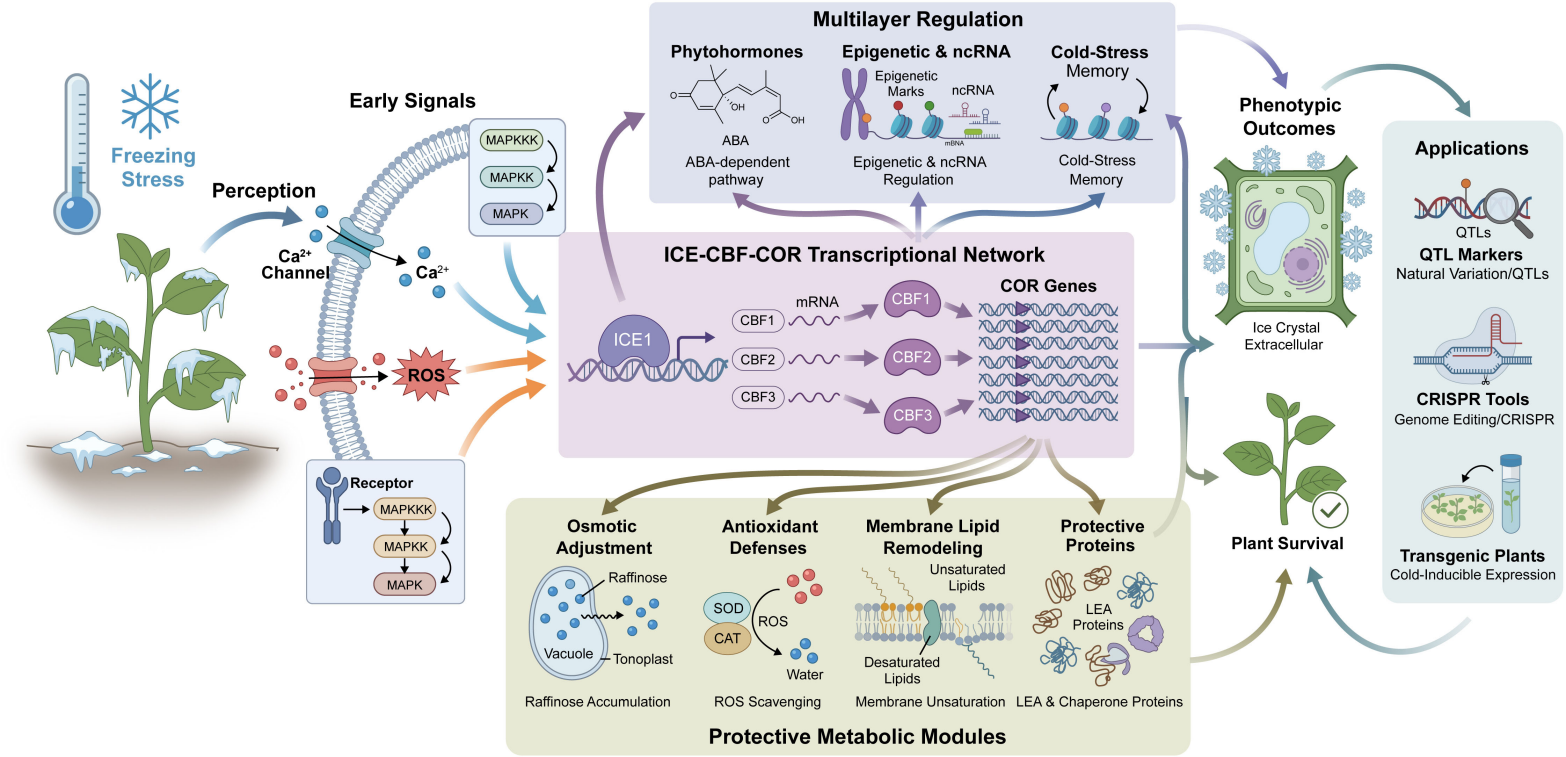
Molecular mechanisms of plant freezing tolerance.

However, major bottlenecks remain in the research and application of cold hardiness and freezing tolerance (Grossman, 2023; Plessis, 2023; Arora, 2025). Inconsistent definitions and phenotyping systems for cold hardiness and freezing tolerance limit comparisons among studies (Arora et al., 2023; van Zuijlen et al., 2023; Larter et al., 2026), and significant differences in experimental paradigms, such as the pre-acclimation state of materials, cooling speed, and whether and how ice nucleation is induced, also make it difficult to reproduce results (Einbock and Conen, 2024; Ralser et al., 2024; Klára et al., 2025; Sun et al., 2025). Although many genes and molecules associated with cold and freezing tolerance have been identified through multi-omics studies, rigorous causal evidence remains insufficient, and key mechanistic links are not always clear (Ding et al., 2024; Kanovska et al., 2024; Mascher et al., 2024; Qian et al., 2025). Here, we adopt a causal framework of “perception – signaling networks – phenotypes – applications” to integrate the molecular mechanisms of plant freezing responses and freezing tolerance, evaluate the strength of evidence for each module, and discuss translational pathways for improving crop cold hardiness (**Figure 1**).

## 2 Physiological and phenotypic assessment framework for freezing stress

Discrepancies exist among laboratories in low-temperature treatment regimes and phenotypic indicators used to assess cold hardiness and freezing tolerance (Mir et al., 2019; Zhang et al., 2022). Key design variables include whether approximately 4°C pre-acclimation is performed (Guo et al., 2023; Xiao et al., 2024), the cooling schedule and rate (Barnes et al., 2023), ice-nucleation procedures, including whether nucleation is spontaneous or deliberately induced, the nucleation temperature, and the use of ice chips, sprayed water, or biological/particulate nucleators (Hamilton et al., 2023; Kobayashi et al., 2024), and the time of sample collection and recovery assessment (Chu et al., 2024; Bao et al., 2025a). Some studies use a slow cooling method, hold the plants for several hours, and then warm them up; the survival rate is taken as the endpoint (Wang et al., 2023a; Zheng et al., 2023; Peng et al., 2024). Others use a rapid temperature drop to estimate parameters, such as the semi-lethal temperature (Bao et al., 2025b). Due to the methodological differences in altering the response amplitude and phenotype expression, direct comparison is limited; to ensure the consistency of phenotyping scales and reports across different studies, standardized phenotyping scales and reporting are required.

Common indicators include survival rate or lethal temperature (whole-plant freezing effect) (Sirooeinejad et al., 2024; Kirchhof et al., 2025); electrolyte leakage (membrane integrity, with larger leakage indicating greater injury) (Kitawaki et al., 2023; Deng et al., 2025); the maximum quantum efficiency of photosystem II (F_v_/F_m_) and the operating efficiency of PSII photochemistry (ΦPSII), two complementary parameters that together reflect low-temperature photoinhibition and the functional status of leaf photosynthetic performance (Ren et al., 2023; Acevedo‐Siaca and McAusland, 2025; Zhao et al., 2025); freezing and supercooling points, ideally estimated from freezing curves, infrared thermography, or differential thermal analysis, and reported as the temperatures at which initial ice formation occurs to indicate freeze-avoidance capacity (Stegner et al., 2024; Vitasse et al., 2025); and physiological/biochemical traits such as malondialdehyde (MDA), reactive oxygen species (ROS), and osmolytes (such as soluble sugars and proline) (Zhao et al., 2022; Jin et al., 2023; Zhang et al., 2023; Wang et al., 2025). In tomato seedlings maintained at 4 °C, the operating efficiency of PSII photochemistry fell from 0.58 after 30 min to 0.46 after 180 min. Over the same chilling period, the maximum quantum efficiency of photosystem II also showed a sustained decline, pointing to a gradual loss of PSII functional integrity under low-temperature stress (Agarwal et al., 2023). Since the maximum quantum efficiency of photosystem II mainly represents the potential upper limit of PSII efficiency, rather than whole-leaf carbon assimilation per se, it is best considered alongside the operating efficiency of PSII photochemistry, electron transport rate, and gas-exchange traits when broader photosynthetic performance and its agronomic relevance are being evaluated (Urzinger et al., 2024; Acevedo‐Siaca and McAusland, 2025). Leaf water potential and osmotic potential provide another useful layer of information, as cold stress and extracellular ice formation can alter plant water relations by reducing external water potential, limiting water uptake, and intensifying cellular dehydration (Delapierre et al., 2024; Li and Hoch, 2024). A more negative osmotic potential typically indicates solute accumulation during osmotic adjustment, helping cells retain water and maintain turgor under dehydration stress. In a recent freezing experiment, leaf water potentials of approximately −0.80 ± 0.64 MPa and −0.57 ± 0.28 MPa were observed under different frost-exposure regimes. These values suggest that water-potential measurements can offer quantitative insight into freezing responses, although they should be interpreted in relation to osmotic adjustment, photosynthetic recovery, and the details of the treatment regime, rather than being treated as fixed injury thresholds on their own (Kane and McAdam, 2025).

Interpretation should take into account the inherent limitations of each assay. Electrolyte leakage, for example, is a useful indicator of membrane injury, but it does not fully capture the overall extent of cold-induced damage. The maximum quantum efficiency of photosystem II reflects the potential upper limit of PSII performance; on its own, however, it cannot adequately describe whole-leaf carbon assimilation, and should therefore be assessed together with the operating efficiency of PSII photochemistry, electron transport, and gas-exchange parameters. Similarly, elevated MDA levels point to oxidative damage, although care is needed to distinguish the direct effects of cold stress from secondary injury processes that develop later. Depending on a single trait may also distort genotype or treatment rankings, particularly when survival is considered without accounting for post-stress recovery and subsequent growth. Accordingly, freezing phenotyping is best conducted under a controlled-freezing protocol, with explicit reporting of the acclimation status, tissue type or developmental stage, starting temperature, cooling rate, ice-nucleation method and temperature, target freezing temperature, exposure duration, rewarming rate, and post-stress recovery period. For meaningful comparison across studies, a core set of indices should be reported where feasible: survival or LT_50_, electrolyte-leakage-based injury or LT_50_, chlorophyll fluorescence traits such as F_v_/F_m_ and ΦPSII, freezing or supercooling point, and selected biochemical or water-relation parameters, including MDA, ROS, compatible solutes, water potential, and osmotic potential. For studies using freezing curves or differential thermal analysis, it should also be made clear whether the freezing program included a deliberately imposed isothermal hold, or whether the recorded response was simply a short-lived exothermic event associated with ice nucleation.

## 3 Cold sensing and early signaling: membrane dynamics, Ca^2+^ and ROS networks

### 3.1 Membrane-based cold perception and candidate sensors

The initial perception of sudden temperature drops is at the subcellular level (Jung et al., 2023). Lipid phase transition in biomembranes at low temperatures reduces membrane fluidity (Huang et al., 2025), and mechanical activation of membrane receptors and ion channels is induced (Ding et al., 2022). Accordingly, changes in the biophysics of the plasma membrane and endomembrane system are often considered to serve as a pathway for cold signals (Xia et al., 2023; Luo et al., 2024). Among the molecular candidates linking membrane-level cold perception to intracellular signaling, COLD1 is one of the more clearly characterized examples in crop plants. It is generally regarded as a membrane-associated cold-sensing or regulatory protein that couples low-temperature perception with G-protein-mediated Ca^2+^ signaling. In rice, the COLD1*–*RGA1 module has been proposed to connect cold-induced membrane perturbation with Ca^2+^ influx. In maize, by comparison, ZmCOLD1 is localized to both the plasma membrane and endoplasmic reticulum, interacts with the G-protein α subunit ZmCT2, and supports Ca^2+^-related chilling tolerance during germination and early seedling development (Lamers et al., 2020; Yan et al., 2022; Zhou et al., 2023). These Ca^2+^ signals are subsequently interpreted by downstream Ca^2+^-dependent signaling modules, thereby contributing to CBF/COR-type transcriptional reprogramming and improved cold or freezing tolerance (Liu et al., 2021). Membrane-skeleton remodeling and changes in cytoskeletal tension may also affect temperature perception (Kumar et al., 2023). In addition, under cold stress, osmotic changes and transmembrane water redistribution will also occur; these changes can be transformed into chilling input signals (Yu et al., 2024).

### 3.2 Ca^2+^ signatures and decoding modules

Although the upstream sensing routes are not necessarily the same, many proposed cold-perception pathways, including *COLD1*-associated signaling and membrane-channel-mediated modules, ultimately converge on transient increases in intracellular calcium ions (Ca^2+^). Cold treatment quickly increases free Ca^2+^ in the cytosol and nucleus, forming a “cold calcium peak” (Dong et al., 2022; Ming et al., 2025). Ca^2+^ serves as a second messenger that transmits information through Ca^2+^ sensors, and genetic or pharmacological inhibition of Ca^2+^ signaling in *Arabidopsis* and rice weakens the induction of downstream cold-responsive genes (Guo et al., 2025). The core sensor modules are calmodulin and calmodulin-binding proteins; Ca^2+^-dependent protein kinases (CDPKs) and the calcineurin B-like (CBL)–CBL-interacting protein kinase (CIPK) network (Allan et al., 2022; Zeng et al., 2023; Xu et al., 2024). In tea, *CsCDPK4* responds to cold-induced Ca^2+^ increases; its knockdown abolishes the cold-tolerance enhancement mediated by volatiles such as (*Z*)-3-hexenol, thereby linking Ca^2+^ signals to cold-tolerance outputs (Liu et al., 2025). In rice, the endoplasmic reticulum-localized calreticulin *OsCRT3* undergoes a conformational change at low temperatures and enhances its interaction with *OsCIPK7*. Activated *OsCIPK7* then interacts with plasma-membrane *CBL7/CBL8* to form a Ca^2+^-signaling complex and activate cold-response pathways (Guo et al., 2023). Consistently, the loss of *OsCRT3* or *OsCIPK7* significantly reduces seedling cold-acclimation ability, while overexpression improves cold tolerance (Wang et al., 2024a; Zeng et al., 2025; Zhang et al., 2026). CIPKs have also been functionally verified in other plants, such as *Camellia* and grapes (Yu et al., 2022; Mao et al., 2023). For example, *CsCIPK11* phosphorylates glutathione S-transferase *GSTU23* to enhance its activity and improve the freezing tolerance of tea (by overexpression) or reduce it (by knockdown) (Di et al., 2024); *CsCIPK20* can enhance cold tolerance by stabilizing the ascorbate-biosynthetic enzyme *VTC1* and increasing antioxidant capacity (Di et al., 2025). In short, these studies suggest that the CBL–CIPK pathway is at the core of cold signal transduction.

### 3.3 ROS burst and kinase cascades in early signaling

After cold perception, reactive oxygen species (ROS) accumulate rapidly. Cold stress disrupts electron transport in mitochondria and chloroplasts, leading to an excess of ROS (Mittler et al., 2022; Wang et al., 2024), and plasma-membrane NADPH oxidases (respiratory burst oxidase homologs, RBOHs) may also be activated to produce extracellular hydrogen peroxide (H_2_O_2_) (Di et al., 2025). At moderate levels, ROS function as signal mediators that trigger antioxidant and defense programs; however, at high concentrations they can cause lipid peroxidation and cellular damage, and therefore need to be tightly regulated. As a result, the appropriate addition of exogenous H_2_O_2_ can enhance the plant’s tolerance to cold stress (Liu et al., 2022), and at this time, antioxidant systems need to be activated to scavenge excess ROS in the cells (Ravi et al., 2023). Ca^2+^ and ROS can form a feedforward loop (Jimenez et al., 2025): Ca^2+^ activates RBOHs, thereby increasing the amount of ROS; in addition, ROS can regulate the entry of Ca^2+^ through Ca^2+^ channels to enhance and extend the cold signal.

**Figure 2:**
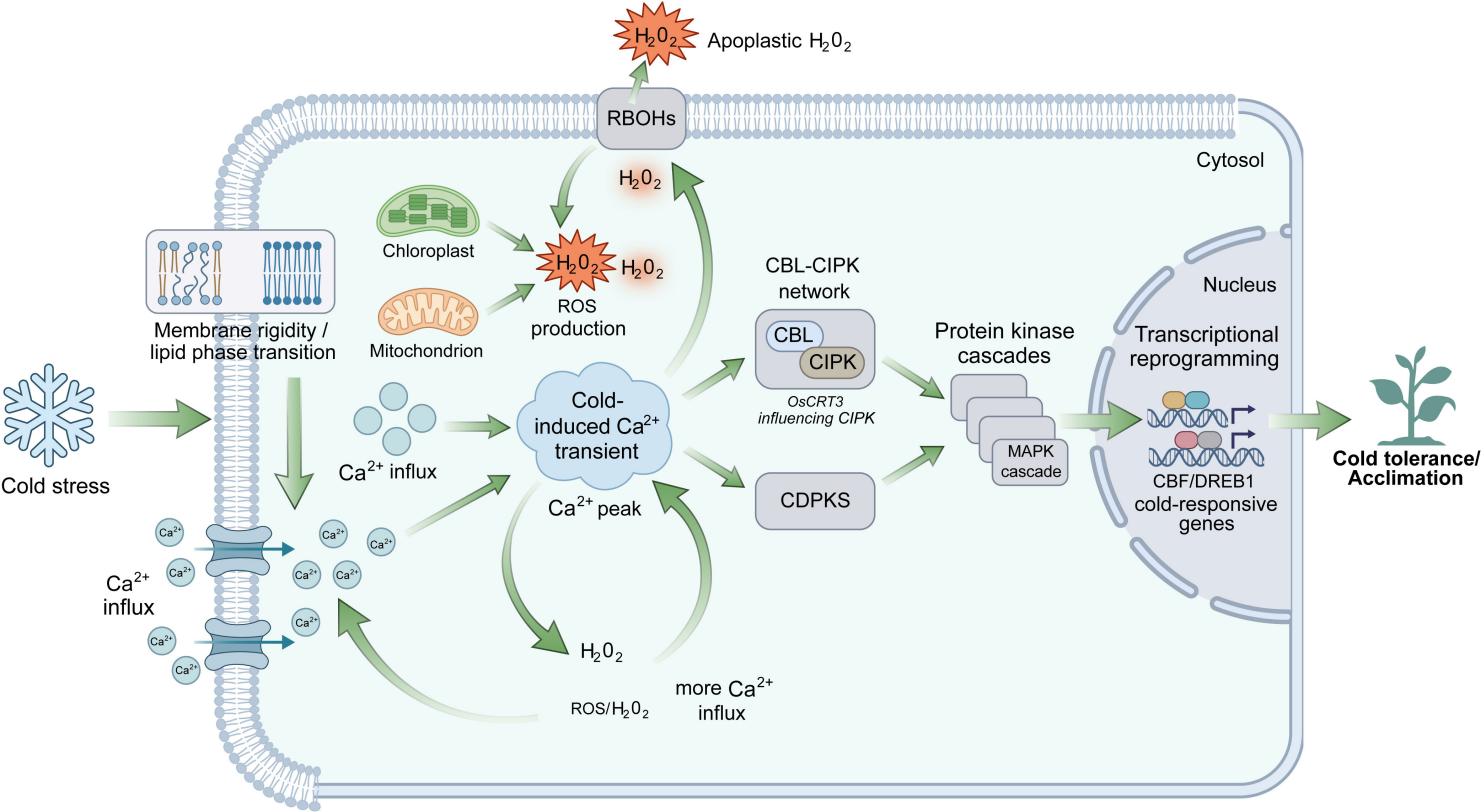
Cold stress triggers Ca^2+^–ROS signaling and transcriptional reprogramming. Cold stress can cause plasma membrane rigidity and induce lipid phase transitions, activating membrane channels to increase Ca^2+^ influx and produce cold-induced Ca^2+^ transients and peaks. Chloroplasts, mitochondria, and the plasma membrane RBOHs stimulate the production of ROS (represented by H_2_O_2_), accompanied by apoplastic H_2_O_2_ accumulation; ROS (e.g., H_2_O_2_) also promotes Ca^2+^ influx, forming a Ca^2+^–ROS positive-feedback loop. In the cytosol, signals are transmitted through the CBL–CIPK network (*OsCRT3* modulates *OsCIPK7*) and CDPKs, which activate kinase cascades that include mitogen-activated protein kinases (MAPKs). These signals are integrated by the nucleus to promote transcriptional reprogramming, and cold-responsive genes such as *CBF/DREB1* are induced to improve the plant’s cold tolerance and cold-acclimation capacity.

The early signal transmits via a multi-level protein-kinase cascade, and the transient physical input is converted into a persistent transcriptional program. In addition to CDPKs and CIPKs, cold stress also activates the mitogen-activated protein kinase module (Manna et al., 2023). In *Arabidopsis*, the cold-responsive *MKK2–MPK4/MPK6* pathway is required for cold tolerance: *mkk2* mutants are highly sensitive to cold, whereas sustained *MPK6* activation increases C-repeat binding factor (*CBF*) expression (Teige et al., 2004; Zhang and Zhang, 2022). In jujube, overexpression of *ZjMAPKK4* enhances cold tolerance and activates the *ZjICE1–ZjCBF* pathway, which may involve *NAC78* (Wang et al., 2025). *OsMPK4* can be activated by cold in rice, then it phosphorylates and stabilizes *MOC1* to enhance the transcription factor’s binding capacity to other factors and thus improve cold tolerance (Li et al., 2026). SNF1-related protein kinase 2 (SnRK2) kinases, such as OPEN STOMATA 1 (OST1), are traditionally located in abscisic acid (ABA) signaling and also participate in cold responses (Zhang et al., 2024; Kang et al., 2025) (**Figure 2**).

Many of the early signaling components are still unlinked to defined primary receptors, and functional validation is uneven. The mechanistic link between membrane phase transitions and initial sensing is still hypothetical, and there is insufficient direct evidence to support it; thus, it is considered a weak-evidence area. Compared with Ca^2+^ signaling and kinase networks, which have extensive *in vivo* genetic data supporting their role in cold tolerance, there is currently a lack of such evidence for the other system. In the future, we need to find real primary thermosensors and clarify their hierarchy, crosstalk, and coupling in parallel signaling pathways.

## 4 Transcriptional regulation of freezing tolerance: *ICE–CBF–COR* axis and co-regulatory networks

### 4.1 Canonical *ICE–CBF–COR* cascade

Transcription factors serve as key interpretive nodes within the cold-response network, linking early Ca^2+^, ROS, kinase-mediated, and hormonal cues to downstream transcriptional reprogramming. By binding selectively to stress-responsive promoter regions, these regulators modulate the expression of *CBF*/*DREB1* and *COR* genes, together with genes associated with osmolyte accumulation, antioxidant protection, membrane lipid remodeling, and the synthesis of protective proteins (Hwarari et al., 2022; Kidokoro et al., 2022; Ding et al., 2024; Kim et al., 2024). Within this regulatory layer, the *ICE–CBF*/*DREB1–COR* pathway is generally viewed as a central cold-responsive module, through which perceived low-temperature signals are converted into protective patterns of gene expression. In general, *ICE* refers to *ICE1/SCREAM*-type basic helix–loop–helix transcription factors; they are stable in the absence of stress but are activated by cold. Activated *ICE* binds to cold-responsive cis-elements in the *CBF* promoter and induces *CBF1*, such as through histone acetylation (Hwarari et al., 2022). *CBF/DREB1* genes encode APETALA2/ethylene-responsive element binding factor-family transcription factors and serve as primary cold-induced regulatory hubs (Qian et al., 2024). Accumulated *CBF* proteins can activate a large number of cold-responsive (*COR*) genes, such as antifreeze proteins, dehydrins and enzymes involved in sugar metabolism, to enhance freezing tolerance (Wang et al., 2024b). First proposed in *Arabidopsis*, this cascade has since been shown to have homologous members across diverse taxa and is widely regarded as the core framework of the cold response (Peng et al., 2025). *CBF* overexpression in *Arabidopsis* significantly enhances freezing tolerance; however, it may cause growth penalties, whereas disruption of the *CBF* pathway reduces cold tolerance, providing causal evidence linking this pathway to low-temperature tolerance phenotypes (Huang et al., 2025).

### 4.2 Tuning of the CBF regulon across species and development

Although it has been conserved through evolution, the regulation of the *ICE–CBF* module shows species differences at various levels, such as tissues and development. Most angiosperms contain *CBF* genes, but the induction and functional effects of *CBF* in tropical monocots such as rice and maize are usually weaker than in cold-tolerant dicots (Kang et al., 2025). In wheat and barley, *CBF* clusters on group-5 homeologous chromosomes exhibit copy-number variation related to differences in cold tolerance (Roeder et al., 2025). In rye, natural allelic variation in *ICE1* may enhance *CBF* activation and has been proposed to contribute to its generally higher cold tolerance compared with wheat (Caccialupi et al., 2023). Based on these patterns, it can be inferred that the evolutionary tuning of the *CBF* module has occurred via gene expansion, cis-element diversification and divergence of upstream regulatory inputs. In addition, tissue-specific cold tolerance can be supported by pathways other than the *CBF* pathway. For instance, in high-altitude rice (plateau japonica), a *CTB5* (homeodomain-leucine zipper, HD-Zip) variant promotes gibberellin (GA) biosynthesis under low temperatures to protect anther development, and upregulates the ABA receptor *PYL9* to alleviate oxidative damage; therefore, this represents coordinated cold tolerance in both vegetative and reproductive organs (Guo et al., 2025).

### 4.3 Co-regulators and parallel transcription factors

Beyond the well-characterized *ICE–CBF*/*DREB1–COR* cascade, other transcription factors also contribute to freezing tolerance, acting through co-activation, transcriptional repression, feedback control, and, in some cases, *CBF*-independent regulatory routes. *CAMTA* proteins can directly induce *CBF* transcription in the early stages of cold exposure, and they are generally regarded as a mechanistic link from Ca^2+^ signaling to transcriptional output (Chao et al., 2022). *MYB* and *WRKY* factors also regulate the modulation of *CBFs*. In tomato, *SlMYB15* promotes the expression of *CBF1/2* by up-regulating the lipoxidase *LOXD* and modulating the jasmonic acid (JA)-related factor *MYC2*; *SlWRKY2* forms a complex with a BTB protein and represses the activity of the *CBF* pathway (Xu et al., 2024; Li et al., 2025a). In apple, *MdbHLH4* interacts with an *ICE1*-like protein and enhances its ubiquitin-mediated degradation, thus reducing the expression of *MdCBF1/3*; overexpression reduces cold tolerance (Yang et al., 2023). Post-translational regulation of *ICE1* is also key. In *Arabidopsis*, the E3 ligase *HOS1* accelerates the degradation of *ICE1* under cold conditions, thereby tempering excessive signals to prevent growth arrest, and rapidly down-regulates the expression of *CBFs* when temperatures rise temporarily to avoid wasteful resource consumption (Larran et al., 2023; Wang et al., 2023b; Yang et al., 2025). SUMOylation (conjugation of the small ubiquitin-like modifier, SUMO) can stabilize *ICE1*; the SUMO E3 ligase *SIZ1* enhances the stability of *ICE1* and maintains *CBF* activation, and the *siz1* mutants exhibit reduced cold tolerance (Fang et al., 2022; Kwak et al., 2024). To summarize, multiple studies have demonstrated that the post-translational regulation pathway involved in the cold-stress response within the *ICE–CBF* module is highly conserved.

**Figure 3:**
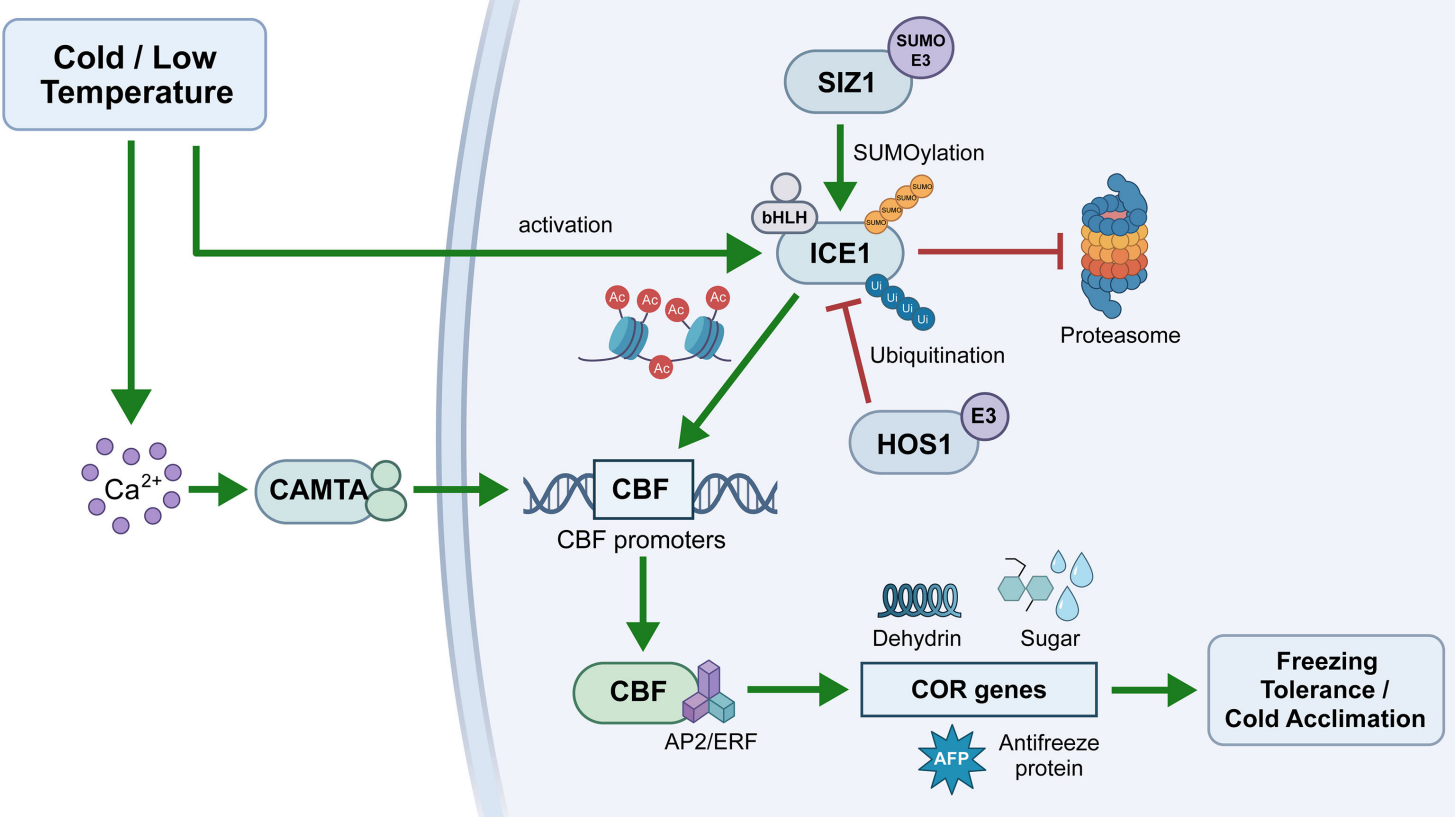
Low-temperature perception and the *ICE1–CBF–COR* transcriptional cascade. Cold stress can cause a Ca^2+^ response and activate *CAMTA*, which binds to the *CBF* promoter. Simultaneously, cold directly activates the basic helix-loop-helix factor *ICE1* to promote *CBF* transcription and chromatin histone acetylation. *ICE1* is post-translationally regulated: The SUMO E3 ligase *SIZ1* mediates SUMOylation, and the E3 ligase *HOS1* mediates ubiquitination and proteasome-mediated degradation. Induced *CBFs* (AP2/ERF family) activate *COR* genes, which in turn promote the synthesis of dehydrins, sugars and antifreeze proteins to improve freezing tolerance and low-temperature acclimation.

Apart from the regulation of *CBF*, many transcription factors can also regulate *COR* genes or other protection pathways directly, forming parallel modules that are associated with the *CBF* axis. In maize, genome-wide association study (GWAS) connects the natural variation of class B heat shock transcription factors, such as *ZmHSF21*, with chilling tolerance at low temperatures during seedling emergence and early stages (Jiang et al., 2022; Li et al., 2022; Gao et al., 2024a). Favorable promoter variants enhance the induction of *HSF21* under low-temperature conditions, and *HSF21* directly regulates lipid-metabolism genes to maintain membrane homeostasis. Notably, this allele enhances cold tolerance with no significant yield reduction. In rice, the tillering and plant-height regulator *Monoculm1* (*MOC1*) has emerged as a cold-response node. Under low-temperature conditions, *OsMPK4* phosphorylates and stabilizes *MOC1*; as part of a ternary complex formed with *OsbZIP79* and *OsNAC5*, it enhances the expression of downstream cold-tolerance genes, such as *OsDREB1G* (Li et al., 2024; Li et al., 2026). The complex can also stabilize *OsbZIP79* and *OsNAC5* to reduce their degradation rates and thereby enhance the output of the signal. Large-scale haplotype analysis shows that there is an enrichment of favorable *OsDREB1G* promoter haplotypes in cold-tolerant lines, supporting a role for natural regulatory variation. Together, these advances expand the *ICE–CBF* framework and indicate that multiple transcriptional modules jointly regulate cold and freezing tolerance (**Figure 3**).

At the transcriptional level, regulatory networks act as an information hub for freezing responses, converting temperature signals into broad-scale reprogramming of gene expression. The *ICE–CBF*/*DREB1–COR* module forms a broadly conserved transcriptional framework, while CAMTA, MYB, WRKY, HSF, bZIP, NAC, and related regulators introduce a degree of flexibility that varies with species, tissue context, and developmental stage. For many recently identified transcription factors, evidence is primarily based on overexpression or loss-of-function phenotypes, and their direct targets and integration into established pathways have not yet been clarified. Conflicts in the seemingly contradictory regulatory roles of some transcription factors in different studies may be due to differences in treatment protocols, functional redundancy or compensation mechanisms; therefore, standardized comparison experiments and cross-species functional complementation need to be carried out to explore these potential causes.

## 5 Metabolic and cellular protection modules under freezing stress

### 5.1 Osmotic adjustment and compatible solutes

Low temperature and extracellular ice formation reduce cellular water availability, thereby promoting dehydration and oxidative stress. To preserve cellular function under these conditions, plants rely on metabolic reprogramming together with multiple layers of protective responses. Species or genotypes with effective osmotic adjustment generally maintain turgor as water potential declines by lowering osmotic potential through the accumulation of compatible solutes. Reported values include leaf water potentials of approximately −0.80 ± 0.64 and −0.57 ± 0.28 MPa under different frost-exposure regimes, as well as an osmotic potential of about −2.2 ± 0.2 MPa in the leaf base of cold-acclimated wheat. Recent studies further indicate that cold tolerance can be strengthened through the accumulation or regulation of soluble sugars, proline, glycine betaine, and putrescine (Ming et al., 2021; Dahro et al., 2022; Li et al., 2022; Qiu et al., 2024; Kane and McAdam, 2025; Stegner et al., 2025). Consistent with this view, a prominent biochemical feature of cold responses is the buildup of compatible osmolytes, including soluble sugars such as sucrose, glucose, and raffinose family oligosaccharides, together with proline, glycine betaine, and polyamines such as putrescine and spermidine (Yan et al., 2022; Wang et al., 2024; Huang et al., 2025; Qu et al., 2025). These compounds support osmotic adjustment and also help preserve cellular integrity by stabilizing proteins and membranes during freeze-induced dehydration. Mechanistically, this response can be attributed in part to colligative freezing-point depression; however, within plant tissues, it more directly reflects osmotic adjustment. By accumulating compatible solutes, cells lower osmotic potential and water activity, reduce the chemical potential of water, and consequently restrict freeze-induced dehydration as well as the movement of water toward extracellular ice. When ice nucleation occurs, the release of latent heat may cause a brief thermal arrest or exothermic signal, whereas a true isothermal hold is expected only when it is deliberately imposed as part of the experimental freezing protocol.

In many cold-acclimated plants, sucrose and raffinose family oligosaccharides increase significantly, accompanied by the induction of biosynthetic genes, such as sucrose synthase and raffinose synthase (Dai et al., 2022; Weiszmann et al., 2023; Berg et al., 2025). In addition, the transgenic expression of the cold-tolerance-associated grape gene *VaSUS2* in *Arabidopsis* and tomato increases sucrose and fructose content, enhances cold tolerance through a decrease in electrolyte leakage, MDA, H_2_O_2_ and an increase in superoxide dismutase and peroxidase activity as well as proline and soluble sugar levels, thereby suggesting that enhanced sugar metabolism and antioxidant capacity contribute to increased cold tolerance (Li et al., 2023). Proline is also a well-recognized osmoprotectant, accumulating predominantly in the cytosol where it reduces osmotic potential, promotes water retention and turgor maintenance, and contributes to the stabilization of proteins and membranes under dehydration caused by extracellular ice formation (Alvarez et al., 2022; Wang et al., 2024). In tomato, *SlWRKY51* directly activates *P5CS1* to enhance proline accumulation under cold stress; loss of *SlWRKY51* leads to reduced proline content and cold-sensitive traits (Wang et al., 2024). Exogenous proline or its precursors can also improve freezing tolerance to some extent (Alvarez et al., 2022; Avendaño et al., 2025). These osmolytes are likely to function in a coordinated manner rather than as isolated protective compounds. Soluble sugars and raffinose-family oligosaccharides support osmotic adjustment while also helping to stabilize proteins and membranes; proline contributes to cellular hydration and redox homeostasis, glycine betaine protects protein*–*membrane complexes, and polyamines may influence membrane charge, ROS buffering, and stress-related signaling pathways. Polyamines and glycine betaine are likewise induced in certain cold-tolerant species, where they may reinforce membrane stabilization and ROS buffering (Blazquez, 2024; Zhou et al., 2025); nevertheless, the causal evidence for these roles remains uneven across compounds and species.

### 5.2 Antioxidant defense and redox homeostasis

Cold stress can also cause an increase in ROS, leading to membrane lipid peroxidation and protein damage; at this time, the level of MDA will rise accordingly. Accordingly, the cold-tolerant genotypes generally have a stronger antioxidant system (Burnett and Kromdijk, 2022; Foyer and Kunert, 2024; Cadena-Ramos et al., 2025), including higher activities of superoxide dismutase, peroxidase, and ascorbate peroxidase, as well as glutathione reductase. As the ascorbate (AsA)–glutathione (GSH) cycle is an essential pathway for degrading hydrogen peroxide (H_2_O_2_), and after cold acclimation, the pools of AsA and GSH have been increased to improve ROS scavenging ability. In tea, *CsCIPK20* enhances cold tolerance by stabilizing the AsA-biosynthetic enzyme *VTC1* via regulation of the *COP9* signalosome, thus increasing AsA content under low temperature and improving antioxidant capacity (Di et al., 2025). Disrupting AsA biosynthesis or inactivating antioxidant enzymes can increase freezing sensitivity (Mishra et al., 2024). Since MDA is a downstream injury outcome that reflects lipid peroxidation, it should be combined with ROS abundance and antioxidant enzyme activities for interpretation (Murphy et al., 2022). Multiple transgenic studies have shown that enhancing antioxidant capacity (e.g., ascorbate peroxidase or superoxide dismutase) improves freezing tolerance, supporting the view that redox protection is positively correlated with cold and freezing tolerance.

### 5.3 Membrane lipid remodeling and integrated protection

**Figure 4:**
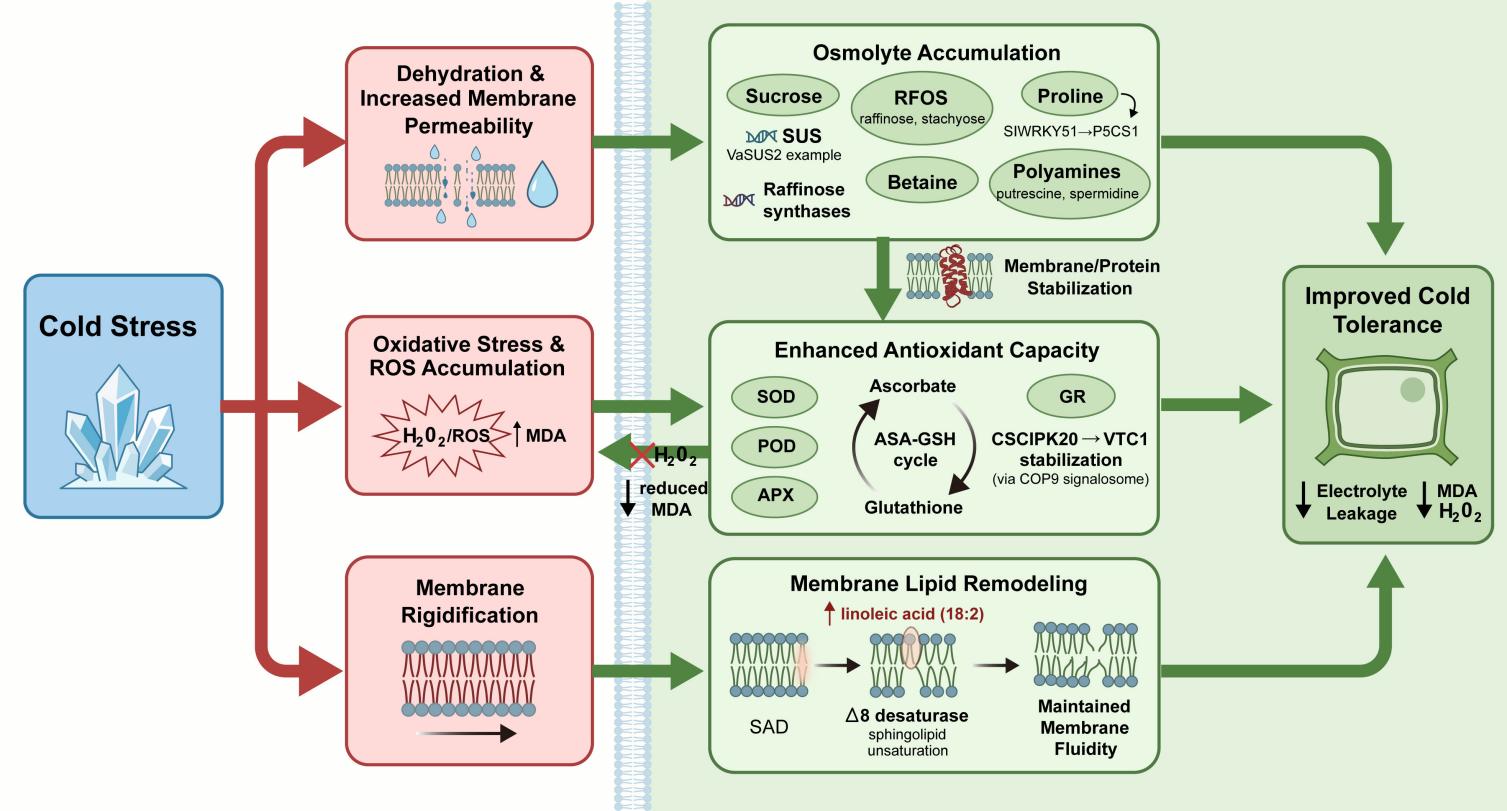
Osmotic adjustment, antioxidant defense, and membrane-lipid remodeling under cold stress. Cold stress leads to cellular dehydration and an increase in membrane permeability, inducing oxidative stress and the production of ROS/H_2_O_2_; MDA is a marker of oxidative damage, and membranes also become rigid. Cold and freezing tolerance are improved through three major protective pathways: (i) osmolyte accumulation (sucrose, RFOs, proline, betaine, polyamines) to stabilize membranes/proteins, as shown by *VaSUS2* and *SlWRKY51* activation of *P5CS1*; (ii) antioxidant reinforcement (superoxide dismutase, peroxidase, ascorbate peroxidase, glutathione reductase and the AsA–GSH cycle), where *CsCIPK20* maintains *VTC1* stability via the *COP9* signalosome pathway, reducing H_2_O_2_ and MDA; (iii) membrane-lipid restructuring (SAD, increased linoleic acid 18:2, and Δ^8^ desaturase-driven sphingolipid unsaturation) to maintain fluidity. Together, these responses reduce electrolyte leakage, lower H_2_O_2_ and MDA content, and enhance cold and freezing tolerance.

Membrane-lipid remodeling is a key protective mechanism underlying cold and freezing tolerance. To maintain adequate membrane fluidity and preserve enzyme activity and transmembrane transport function in cells at low temperatures, a specific proportion of unsaturated lipids needs to be retained. Plants often lower the membrane phase-transition temperature through increased lipid unsaturation (Pan et al., 2024), and introducing more fatty-acid double bonds can delay membrane rigidity at subzero temperatures (Chong et al., 2025). Mutants of the long-chain base Δ^8^ desaturase (*sld1-1*) in *Arabidopsis* exhibit reduced membrane fluidity and impaired growth under cold stress, suggesting that increased sphingolipid unsaturation is beneficial for cold acclimation and tolerance (Huang et al., 2025; Xiao et al., 2026). Maize lipidomics shows that chilling-tolerant lines generally have a higher accumulation of unsaturated fatty acids, and the expression of key enzymes such as stearoyl-ACP desaturase (SAD) is upregulated (Gao et al., 2024b). Changes in certain lipid classes, such as phosphatidylcholine and sphingolipids, are also related to cold tolerance (Angkawijaya, 2024; Kenchanmane Raju et al., 2024). After cold acclimation, the content of linoleic acid (18:2) in the membrane has been observed to be higher than before cold acclimation across multiple phospholipid classes; meanwhile, the quantity of saturated fatty acids will decrease to maintain membrane stability and normal cell function under low temperature conditions (**Figure 4**).

### 5.4 Protective proteins, organelles, and evidence validation

Organelles and proteins are the main targets of protection in freeze-tolerant organisms. At low temperatures, proteins tend to misfold and aggregate; thus, plants mitigate this damage by expressing stress-induced proteins such as molecular chaperones. Dehydrins and late embryogenesis abundant proteins are typical representatives of the *COR* family, which are highly accumulated under conditions of dehydration and freezing (Szlachtowska and Rurek, 2023). Because these proteins are highly hydrophilic and help enzymes remain in a hydrated environment during cellular dehydration, irreversible enzyme damage due to water loss can be minimized. In woodland strawberry (*Fragaria vesca*), the expression level of cold-related dehydrin *Xero2* is positively correlated with cultivar cold hardiness; its overexpression can improve the cold tolerance of tobacco cells (Kanbar et al., 2024). Antifreeze proteins attach to the surface of ice crystals and prevent them from growing (Tas et al., 2023; Obadi and Xu, 2024); in plant research, it has been verified that antifreeze proteins can reduce the harm caused by extracellular ice (Wu et al., 2023; Dong et al., 2025). In addition, heat shock proteins assist in preserving proteostasis during cold stress. HSP70, HSP90, and other chaperones are induced by low temperatures to restore damaged proteins and prevent aggregation (Luklova et al., 2025; Wei et al., 2025). In tomato, through the JA-mediated cold-tolerance pathway, small chaperones (such as HSP17.7) are induced to enhance protein stability (Zhang et al., 2025). Protection of mitochondria and chloroplasts is also needed because cold can cause an increase in ROS accumulation and membrane permeability in these energy organelles (Selinski et al., 2024). Plants enhance their mitochondrial antioxidant capacity and increase the unsaturation of chloroplast membrane lipids, among other responses (Murray and Graether, 2022; Zu et al., 2023; Hernández et al., 2024; Hou et al., 2024; Legen et al., 2024). Some dehydrins relocalize to chloroplasts under cold conditions, which may directly stabilize thylakoid membranes and photosystem complexes.

Omic associations under cold stress need to be distinguished from functional causality. Large fluctuations in metabolites and protective proteins can only be assigned functions if genetic manipulation directly demonstrates a connection to cold tolerance. Proline and dehydrins have supporting evidence from mutant and transgenic lines; on the other hand, some recurrent metabolic changes (such as the accumulation of specific secondary metabolites) in cold-tolerant genotypes lack functional validation and are more suitable as correlation markers. Based on genetic-intervention evidence, freezing tolerance can be summed up in four functional modules: osmolyte accumulation, antioxidation, membrane-lipid reconfiguration and protective proteins; together they help maintain cell survival under freezing stress. The key deficiencies are that the quantitative contributions of each module to overall plant hardiness have not yet been addressed, and the shared regulation or trade-offs among modules remain unclear. The proposition that breeding should balance multiple modules while avoiding metabolic burdens from over-amplifying a single pathway still needs to be verified by multi-gene pyramiding and field improvement trials.

## 6 Regulatory crosstalk and stress memory: phytohormones, epigenetics, and non-coding RNAs

### 6.1 ABA-centered signaling and hormone crosstalk

The low-temperature response is often accompanied by phytohormone signaling, and abscisic acid (ABA) usually serves as an important regulatory component within this framework. Cold exposure can moderately increase the content of ABA and activate canonical pathways, such as the PYL receptor and *SnRK2* kinase pathways, to regulate a group of stress-responsive genes (An et al., 2023; Li et al., 2024). ABA signaling also crosstalks with the *ICE–CBF* pathway: ABA can upregulate specific *CBF* and *COR* genes, whereas *CBFs* can reciprocally regulate ABA-pathway components (Hu et al., 2024). In apple, the ABA-responsive bZIP factor *MdABI5* represses the expression of *MdCBF3* and *MdJAZ*, reducing cold tolerance; conversely, TCP transcription factors, together with HDACs, can restrain *ABI5*-dependent repression and thus improve cold tolerance (Deng et al., 2026). Excess ABA often accompanies growth inhibition, so plants have reduced sensitivity to maintain normal growth under environmental stresses by modulating the responsiveness of ABA. Under freezing conditions, *SnRK2.6/OST1* not only participates in part of the ABA response but also phosphorylates *HAT1* and promotes its degradation via the *HOS1* pathway, thus releasing the inhibition on *CBF* and related genes by *HAT1*. The *OST1–HOS1–HAT1* module enables moderate ABA activation in response to cold stress and prevents maladaptive overactivation (Kavi Kishor et al., 2022; Kang et al., 2025). ABA also helps in the development of cold-acclimation, such as leaf abscission and dormancy (Yang et al., 2023). In addition, the differences and sensitivities of ABA in crops are closely related to their cold tolerance; therefore, it also serves as an essential positive regulator in the process of cold acclimation and freezing tolerance.

Hormonal crosstalk during cold acclimation is best understood as a shifting balance between signals that promote stress protection and those that regulate growth. ABA and JA typically strengthen *CBF*/*COR* activation, antioxidant capacity, and osmotic protection, while GA, ETH, and CK are often reprogrammed in ways that limit growth or help maintain meristematic and reproductive functions. SA and BR add further complexity to this regulatory balance, with their effects depending strongly on dosage, tissue context, and environmental conditions (Waadt et al., 2022; An et al., 2023; Chu et al., 2024; Hu et al., 2024; Wang et al., 2024c; Guo et al., 2025; Khan, 2025). These interactions appear to proceed through several representative routes, such as ABA*–CBF*/*COR* coupling, JA-mediated release or reinforcement of *ICE*/*CBF* activity, BR*–*SA balancing via *TaSAMT1*, and GA/ABA-mediated protection that varies among tissues. Jasmonic acid (JA) usually rises early after cold treatment and regulates defense responses as well as redox balance. In tea, *CsLUX/ARRHYTHMO* was proposed to buffer JA homeostasis by repressing the JA-biosynthetic gene *CsLOX2* and to modulate JA signaling via interacting with *CsJAZ1*, thereby weakening *CsJAZ1*-mediated repression on *CsICE1* and maintaining an appropriate cold response (Ding et al., 2024; Wang et al., 2024c). Naturally occurring loss-of-function alleles of *CsLUX* lead to a JA over-accumulation response to cold and reduced tolerance, indicating that JA has a dose-dependent effect on cold acclimation and freezing tolerance. Salicylic acid (SA) also has context-dependent effects: Moderate SA can induce the expression of pathogenesis-related genes and antioxidant defenses to enhance cold tolerance, but excessive SA can inhibit growth and antagonize ABA signaling (Mittler et al., 2022; Ortega et al., 2024; Li et al., 2025b). *TaSAMT1* in wheat is an SA-to-methyl salicylate (MeSA) methyltransferase that reduces free SA; overexpression of *TaSAMT1* improves freezing tolerance, while knockout increases SA accumulation and cold sensitivity (Chu et al., 2024). *TaSAMT1* links brassinosteroid (BR) and SA pathways: Under cold conditions, BR signaling upregulates the expression of *TaSAMT1* by increasing histone acetylation, thereby activating it; this reduces SA levels and achieves a balance between BR-positive and SA-negative regulation of cold tolerance (An et al., 2023; Sanchez-Munoz, 2024).

Gibberellins (GA) mainly promote growth, and the level of GA is often reduced during cold acclimation, causing plants to switch to a state that restrains growth but enhances freezing tolerance (Zhu et al., 2022). Notably, the upland rice allele *CTB5* maintains GA biosynthesis under cold conditions to protect anther development and increase ABA content in vegetative tissues to reduce chilling/freezing injury, indicating tissue- and stage-specific hormone allocation (Guo et al., 2025). Ethylene (ETH) and cytokinins (CK) show a more complex pattern: elevated ETH/CK activity generally favors growth, although it is often linked to reduced stress tolerance. Therefore, ETH/CK signaling is often suppressed under cold conditions, whereas JA/ABA-related stress signaling often increases (Waadt et al., 2022). However, some CK activity may be needed to keep the meristem functional under low-temperature growth (Argueso and Kieber, 2024; Yang et al., 2026). Brassinosteroids (BR) can improve cold tolerance by priming, but excessive BR can cause damage to cell membranes during phase transitions of lipids, which is also dose-dependent (Hu et al., 2022, Hu et al., 2023; Yueshan et al., 2024). ABA and CK often have opposite effects; the increase in ABA related to cold acclimation and the decrease in CK may partly cause the growth inhibition under cold conditions (Waadt et al., 2022; Jarošová et al., 2024). Taken together, freezing stress appears to remodel hormone homeostasis rather than trigger a single, linear signaling pathway. Stress-related hormones, particularly ABA and JA, generally strengthen protective transcriptional programs, antioxidant capacity, and osmotic adjustment, whereas growth-related signals such as GA, ETH, and CK are recalibrated to balance survival with continued growth and organ development. Since these pathways converge on shared transcription factors, redox regulators, and metabolic modules, hormonal crosstalk represents an important regulatory layer that connects cold perception with downstream freezing-tolerance outcomes. Most studies have focused on a pair of interactions (for example, ABA–JA, ABA–ETH), and a systematic dissection of higher-order, multi-hormone circuitry remains limited (Khan, 2025). Combining higher-order mutants and hormone reporters with single-cell spatiotemporal profiling should help resolve both the hierarchy and the signal specificity of hormonal crosstalk during cold responses. Representative studies on phytohormone-mediated regulation of plant cold hardiness are summarized in **Table 1**.

**Table 1:** **A** review of studies on phytohormone-mediated regulation of plant cold hardiness.

Species	Cold scenario	Hormone class	Key factor	Main regulatory mechanism	Effect	Ref
*Malus domestica*	F	ABA	*MdHDA6 MdTCP15 MdABI1 MdCOR47*	*MdHDA6* represses MdTCP15 via histone deacetylation limiting ABI1 and restoring COR47 transcription	+	Guo et al. (2023)
*Triticum aestivum*	F	BR–SA	*TaSAMT1 TaBZR1 TaHAG1* MeSA	*TaBZR1* with TaHAG1 activates TaSAMT1 elevating MeSA and strongly improving freezing survival	+	Chu et al. (2024)
*Arabidopsis thaliana*	F	SL	*MAX2 WRKY41 SMXLs CBFs*	Strigolactone signaling via MAX2 degrades WRKY41 releasing CBF expression and enhancing freezing tolerance	+	Wang et al. (2023a)
*Solanum lycopersicum*	C	JA	*SlSGT2 SlSGT1* steryl glycosides JA	Higher steryl glycoside ratio stabilizes plasma membrane and primes jasmonate CBF responses	+	Deng et al. (2025)
*Camellia sinensis*	C	CK	*CsUGT71A60* cis-zeatin ARR	*CsUGT71A60* glycosylates cis-zeatin to maintain cytokinin balance boosting antioxidant osmoprotection during chilling	+	Zhao et al. (2025)
*Camellia sinensis*	FT	ABA	*CsADH2* (Z)-3-hexenol UGT85A53 ABA	Cold-induced (Z)-3-hexenol activates UGT85A53 enhancing ABA glucosylation and stress integration	±	Jin et al. (2023)
*Arabidopsis thaliana*	C	JA	*COI1 MYC2 SLD1* LCB	Jasmonate COI1 MYC2 activates SLD1 raising LCB unsaturation to sustain membrane fluidity	+	Huang et al. (2025)
*Oryza sativa*	C	JA	*miR1320* PHD17 JA JAZ	*miR1320* represses PHD17 limiting jasmonate biosynthesis and signaling thereby improving rice chilling tolerance	−	Wang et al. (2024a)
*Capsicum annuum*	C	ABA	*CaSnRK2.4 CaNAC035 CaNCED3* ABA	*CaSnRK2.4* phosphorylates CaNAC035 enabling activation of CaNCED3 CaAAO3 and ABA biosynthesis during chilling	+	Zhang et al. (2024)
*Oryza sativa*	FT	ABA–GA	*CTB5 OsHox12 PYL9 OsGA2ox6*	*CTB5* activates GA homeostasis genes and PYL9 to protect anthers and seedlings under cold	+	Guo et al. (2025)
*Solanum lycopersicum*	C	JA	*SlMYB15 SlLOXD SlMYC2 SlCBF1*	*SlMYB15* primes SlLOXD jasmonate biosynthesis then SlMYC2 activates CBF1 CBF2 expression during chilling	+	Li et al. (2025a)
*Solanum lycopersicum*	C	JA	*SlMYB13 SlMYC2 SlHSP17.7* MeJA	Jasmonate-induced SlMYB13 activates SlMYC2 and SlHSP17.7 transcription to enhance chilling tolerance	+	Zhang et al. (2025)
*Solanum lycopersicum*	C	ABA–BR	*DWF BZR1 NCED1* ABA	*BZR1* binds NCED1 promoter elevating ABA biosynthesis enabling brassinosteroid induced chilling tolerance	+	An et al. (2023)
*Solanum lycopersicum*	C	ABA	*ERF15 CBF1 WRKY6* ABA	*ERF15* binds CBF1 and WRKY6 promoters activating ABA-dependent cold-responsive transcription in tomato	+	Hu et al. (2024)
*Pyrus pyrifolia*	FT	ABA–GA	*PpyABF3 PpyWDR5a DAM4 GA2OX1*	*ABF3* recruits WDR5a COMPASS raising H3K4me3 at DAM4 and GA2OX1 to maintain dormancy	±	Yang et al. (2023)
*Camellia sinensis*	F	JA	*CsLUX CsLOX2 CsJAZ1 CsICE1*	*CsLUX* represses LOX2 modulating jasmonate and disrupts JAZ1 binding to ICE1 enhancing freezing tolerance	+	Wang et al. (2024c)
*Arabidopsis thaliana*	C	SA	*Irp9* SA COR	Elevated salicylic acid suppresses COR gene transcription causing temperature dependent growth inhibition	−	Ortega et al. (2024)
*Oryza sativa*	C	CK	tZ ABCG18	Root-derived trans-zeatin via ABCG18 boosts glycolysis TCA raising ATP and energy charge	+	Yang et al. (2026)
*Prunus persica*	FT	BR	EBR PpHDT1 PpDWF4 PpBZR1	EBR induces PpHDT1 repressing PpDWF4 and activating PpBZR1 to reduce chilling injury	+	Hu et al. (2023)
*Prunus persica*	FT	BR	EBR PpCBF5 PpPLD PpLipase	EBR elevates PpCBF5 repressing PpPLD and PpLipase to maintain membrane lipid stability	+	Hu et al. (2022)
*Cucumis sativus*	C	BR	CSN BZR1 CsICE CsCBF	CSN activates brassinosteroid synthesis and signaling rapidly inducing ICE CBF COR genes under chilling	+	Yueshan et al. (2024)
*Oryza sativa*	FT	ABA–JA	ABA JA SA tZ	Leaf cooling increases jasmonate and salicylic acid while root cooling increases abscisic acid	±	Jarošová et al. (2024)

Low-temperature scenario types: C is chilling (non-freezing low temperature), F is freezing (ice formation and freezing stress), FT is complex scenarios (freeze–thaw cycles, overwintering and bud-break stages, organ-specific directional cold stress, postharvest chilling injury, and related conditions). Effect on cold hardiness or cold tolerance: +, enhanced; −, weakened; ±, context-dependent, usually varying with genetic background, developmental stage, hormone dosage, or treatment scenario. SL, strigolactone(s); tZ, trans-zeatin; TCA, tricarboxylic acid cycle; ATP, adenosine triphosphate; MeJA, methyl jasmonate; MeSA, methyl salicylate; EBR, 24-epibrassinolide; LCB, long-chain base; CSN, sodium nitrophenolate.

### 6.2 Epigenetic reprogramming and stress memory

Low-temperature stress can cause epigenetic reprogramming, thereby regulating the expression of genes and forming a “memory” of stress; cold acclimation often occurs simultaneously with changes in histone modifications and DNA methylation. Vernalization in *Arabidopsis* is a classic case of long-term cold memory: prolonged cold repression of *FLC* through H3K27me3 deposition and related silencing allows for flowering competence (Nielsen et al., 2024; Montez et al., 2025). Epigenetic changes also occur during freezing tolerance outside of vernalization, but they tend to be temporary. Active marks such as H3K4me3 and H3 acetylation increase at the promoters of many cold-responsive genes when exposed to cold, and these changes typically disappear after recovery (Wang et al., 2024), which is consistent with their role in the regulation of a short-term response rather than long-term memory. Some studies have shown that brief cold stress or acclimation can enhance the subsequent tolerance in a short time (“cold-stress memory”), which may be related to histone modification and centromere repositioning (Harris et al., 2023; Liu et al., 2023; Wang et al., 2024). In polyploid crops, such as wheat, cold-induced epigenetic states have been reported to be partially inherited by offspring; however, the stability and repeatability of this phenomenon need further rigorous confirmation (Ramakrishnan et al., 2022; Cao and Chen, 2024; Maric, 2024; Niu et al., 2024).

DNA methylation can serve as compelling causal evidence for rice *ACT1*. Multigenerational selection under low temperature can produce inheritable cold-tolerant lines, and the *ACT1* promoter has acquired a stable hypomethylated epiallele that can maintain *ACT1* induction at low temperatures and enhance cold tolerance. CRISPR/dCas9-TET-mediated targeted demethylation demonstrated that promoter hypomethylation causally contributes to increased cold tolerance (Ali et al., 2025; Song et al., 2025). This example indicates that a cold environment can produce heritable epigenetic marks. However, similarly stable and large-effect cold-associated epialleles are uncommon across species. Additional evidence suggests that DNA demethylases and histone-modifying enzymes are also involved in cold responses: disruption of a DNA demethylase in maize disrupts transcriptional homeostasis and reduces cold tolerance, and in wheat, the histone acetyltransferase *TaHAG1* contributes to the activation of *TaSAMT1*, forming an epigenetic node associated with cold hardiness (Liu et al., 2022; Xu et al., 2022; Chu et al., 2024; Ma et al., 2025). Considering the inherent plasticity of epigenetic marks, “memory breeding” has been proposed using chemical epigenetic modulators or epigenome editing to steer epigenetic states (Cheng et al., 2024; Park et al., 2024; Dvorak Tomastikova and Pecinka, 2025; Wang and Bart, 2025; Wang et al., 2025). However, practical application needs to consider the spatial-temporal specificity and developmental effects of the modification, limiting it to the target site and cell type.

### 6.3 NcRNA regulation and integrated memory network

**Figure 5:**
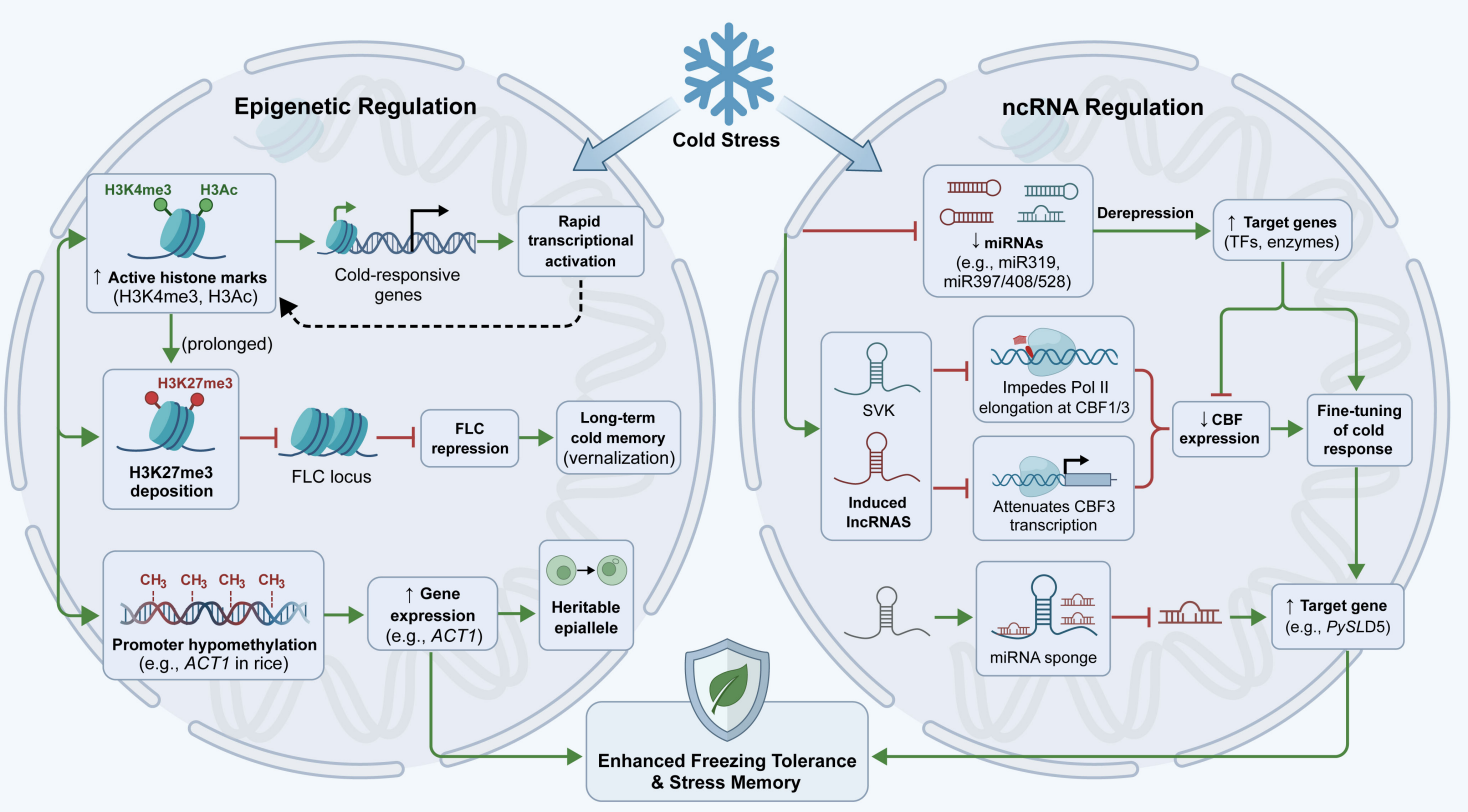
Epigenetic and ncRNA-mediated freezing-stress memory under cold stress. Cold stress engages nuclear epigenetic pathways and ncRNAs to modulate freezing tolerance and establish stress memory. Active histone marks (H3K4me3 and H3Ac) increase, promoting the expression of cold-responsive genes and enabling quick reactivation. Prolonged cold induces H3K27me3 deposition at the *FLC* locus, repressing *FLC* expression and establishing a long-term cold memory (vernalization). At the same time, promoter hypomethylation (such as rice *ACT1*) increases expression and can also produce inheritable epialleles. Reduced miRNAs (*miR319* and *miR397*/*408*/*528*) release inhibition by targeting transcription factors and enzymes. Cold-induced lncRNAs (including *SVK*) inhibit RNA polymerase II elongation at *CBF1/3* and reduce *CBF3* transcription, fine-tuning responses through limiting *CBF* output; miRNA sponges also derepress targets and increase expression (e.g., *PySLD5*).

Non-coding RNAs (ncRNAs) are divided into three major categories—microRNAs, long non-coding RNAs (lncRNAs), and circular RNAs—and their expression levels change in plant responses to low-temperature stress. Cold stress reprogramming is mediated by a large number of microRNAs (miRNAs) and target modules, and some miRNAs directly regulate transcription factors or metabolic enzymes to produce a substantial phenotypic effect. In rice, *miR319* and other miRNAs help improve cold tolerance by participating in the regulatory processes during cold stress; therefore, overexpression or knockout of these specific small RNAs can enhance or reduce plant cold tolerance. A recurring phenomenon is miRNA down-regulation accompanied by target up-regulation, which is related to improved tolerance (Chen et al., 2025; Zhao et al., 2025). Given that individual miRNAs frequently modulate numerous targets, it is generally unclear which genes are specifically associated with phenotype alterations. A group of miRNAs can be regulated concurrently, thereby functioning in combination. Hong and colleagues co-overexpressed the Cu-related *miR397/408/528* trio in rice and maize, generating transgenic lines with significantly enhanced cold and drought tolerance without obvious yield penalties (“3-miR stacking”), indicating the potential of small RNAs for crop stress improvement (Hong et al., 2024; Lyu, 2024). In *Arabidopsis*, the cold-induced lncRNAs *SVALKA* (*SVK*) and *SVALNA* (*SVN*) regulate *CBF* expression in different ways: *SVK*-derived transcripts inhibit RNA polymerase II elongation at *CBF1/3* and can act *in trans* to affect chromatin; *SVN* acts *in cis* near *CBF3* to reduce transcription. The *svk/svn* double mutant exhibits increased *CBF1/3* expression and a modified freezing-tolerance phenotype, indicating that lncRNAs can help fine-tune the cold response (Zacharaki et al., 2023; Rosenkranz et al., 2025). LncRNAs can also serve as miRNA “sponges”; in poplar, two lncRNAs compete for binding to *miR6476*, increase the expression of the DNA replication gene *PySLD5*, and enhance cold tolerance (Yang et al., 2026). Continuous research on ncRNAs may reveal that some are not only involved in protein synthesis, but also have other roles in regulating the process, thereby explaining the differences in cold tolerance among developmental stages and cell types (**Figure 5**).

Although there has been significant progress, many inferences about hormone–epigenetic–small RNA regulation are still based on correlations and limited functional verification. Key gaps cluster in three areas: (i) high-resolution spatiotemporal dynamics *in vivo*, such as live reporters of hormones or epigenetic states; (ii) tissue- and cell-type-resolved mechanisms, because whole-plant measurements cannot reveal local fate decisions; (iii) field-level validation to determine whether the phenotypes observed in the laboratory can be maintained in a complex environment. It remains to be determined whether epigenetic and ncRNA pathways can support stress “memory” across different developmental stages or even generations, and how such plasticity can be utilized in breeding without introducing unpredictable metabolic costs. Beyond the mechanistic progress, the standardization of cross-platform datasets and the development of multi-omics integrative models are expected to build a systems-level framework for how hormone–epigenome–gene networks work together to influence freezing adaptation.

## 7 Translation and outlook: from mechanisms to breeding and engineering

Translating freezing-tolerance and cold-hardiness mechanisms into crop improvement requires the integration of genetic diversity and modern breeding technologies. Large-scale quantitative trait locus mapping and genome-wide association studies have identified many candidate loci related to cold tolerance in crops, such as cucumber, maize, and rice (Das et al., 2022; Li et al., 2022; Dong et al., 2023; Yang et al., 2023; Li et al., 2024; Li et al., 2025; Xu et al., 2025). Whole-genome haplotype analysis and functional verification show that natural allelic differences at these loci can improve low-temperature adaptability, consistent with known cold-tolerance signaling pathways. Some advantageous haplotypes of specific genes in maize, rice and wheat have been found that enhance cold tolerance with minimal or no yield reduction (Yang et al., 2023; Gao et al., 2024a; Gan et al., 2025; Del Pozo et al., 2026). Therefore, systematic exploration of natural variations combined with mechanism-based dissection can simultaneously verify breeding values and provide implementable targets for the development of cold-tolerant varieties.

Breeders have begun using molecular techniques and genetic engineering technologies, and through breeding, they are starting to apply them in agricultural production. Marker-assisted selection can pyramid multiple beneficial alleles, and genome-editing tools such as CRISPR/Cas can knock out negative regulators or reprogram key cis-regulatory elements to enhance the expression of cold-inducible protective programs without adding foreign DNA. Compared with traditional screening of winter ecotypes, these approaches can accelerate the improvement of cold hardiness; however, potential effects on important agronomic traits, such as flowering time and growth duration, must be carefully evaluated. Organ-specific or inducible expression schemes can help reduce growth inhibition caused by the continuous activation of defense pathways. Improvement programs also need to determine the trade-offs between increased cold tolerance and yield, as well as any interactions among these stresses, to avoid improvements in one trait that reduce drought or disease resistance. Future studies should determine which cells respond primarily to cold-sensing components; clarify how different types of cells produce a response when stimulated by various elements individually or jointly; and elucidate the combined effects of multiple factors on these reactions. By standardizing the phenotyping of cold hardiness, the cross-study comparability will be enhanced, and the transition from laboratory discovery to field application will be promoted; thus, crops with broad cold hardiness that can buffer low-temperature risks under conditions of increased climate variability will be realized.
